# The Fitness Barometer: A Best Practice Example for Monitoring Motor Performance With Pooled Data Collected From Practitioners

**DOI:** 10.3389/fpubh.2021.720589

**Published:** 2021-12-10

**Authors:** Tanja Eberhardt, Klaus Bös, Claudia Niessner

**Affiliations:** Institute of Sports and Sports Science, Karlsruhe Institute of Technology, Karlsruhe, Germany

**Keywords:** assessment, monitoring, physical fitness, youth, children

## Abstract

**Introduction:** Motor Performance (MP) in children is an important resource for their future active lifestyle and health. Monitoring of MP is crucial to derive information of trends and to implement specific programs on the base of current MP levels. A variety of MP assessment tools exist, making it difficult to determine a “gold-standard” for assessment and to compare the findings. In Germany, the German Motor Test 6–18 (GMT 6–18) and Kinderturntest Plus 3–10 (KITT+ 3–10) are widely used MP assessment tools. The aim of this paper is to show which key questions can be answered within the context of a best practice example of a MP assessment tool and what can be derived from this for a practical application (the Fitness Barometer).

**Methods:** The raw data of the Fitness Barometer was collected with the MP assessment tools GMT 6–18 and KITT+ 3–10 from 2012 through 2020. Data was pooled anonymously with the e-Research infrastructure MO|REdata and categorized into percentiles for MP and BMI. Overall, we included data of 23,864 children for the statistical analyses. *T*-tests for independent samples, percentage frequency analysis, descriptive statistics (chi- square-test) and single analysis of variance were conducted.

**Results and Discussion:** Children tested reached a mean value of 57.03 (*SD* = 18.85). Of the sample, 12.7% children were overweight or obese and there is a significant difference between age groups [$\chi_{\left(4\right)}^{2}$ = 178.62, *p* < 0.001, Cramer *V* = 0.09; *n* = 23.656]. The relationship between BMI category and mean value of MP was significant [*F*_(4,19,523)_ = 224.81, *p* < 0.001]. During 2020, the year of the COVID-19 pandemic, mean value of endurance and speed decreased [Welch's *F*_(1,573)_ = 8.08, *p* = 0.005; Welch's *F*_(1,610)_ = 35.92, *p* < 0.001]. The GMT 6–18 and KITT+ 3–10 are valid, objective, reliable, and economic MP assessment tools for monitoring MP levels and derive added practical value. Specific programs and interventions should focus on the findings of these. The Fitness Barometer is a best practice example how a standardized assessment tool of monitoring MP point to trends on which practical evidence-based suggestions can be derived with many various partners and expertise.

## Introduction

Systematic monitoring is used in many areas of life to document trends and changes in society and to describe their course over a certain period. For example, a popular monitoring instrument for examining school capacity is the international PISA study (1). The results provoked changes and adjustments in national education plans and policy, and the implementation of specific programs and interventions.

Similarly, identifying the current level of Motor Performance (MP) is crucial to plan, design, and evaluate adequate interventions and programs to maintain and increase physical activity in youth^1^.

MP is one of the main resources for an active lifestyle and therefore of great significance for healthy development in childhood, adolescence, and throughout one's entire lifespan (5–9). The development of MP either encourages or discourages an individual to engage in physical activity through limiting one's opportunities (7, 10–12). With increasing MP, the risk of overweight decreases and maximal oxygen intake in childhood is improved (10, 13). A positive association has been found for cardiorespiratory and muscular fitness (14). Moreover, well-developed MP is related to a lower risk for current and future diseases, and has a positive effect on the physical self- concept development of personality and cognitive abilities (15–18).

Systematic monitoring requires validated, objective, and reliable tools.

There are numerous tools for assessing MP in children and adolescents, and also many theories behind them. However, there is no “gold-standard” through research and the challenge is to bring together various interests (19–21). With quantitative assessment tools, MP levels are measured according to outcome and the results are compared with those of a norm group. In contrast, qualitative assessment tools focus more on how the specific task and its components are performed (22, 23). Additionally, the characteristics and circumstances of the target group, meaning geographical region, socio-economical background, and culture of the participants, determine which assessment tool is preferred (19, 20).

Another challenge of assessing MP is the different purposes of practice and research with the common aim of monitoring MP in children and adolescents and making statements about trends and development. MP assessment tools should meet the general research criteria of objectivity, reliability, and validity. For practitioners, using MP assessment tools and interpreting their results should be simple and easy, and the implementation should be feasible for the specific setting (24).

Comparable monitoring of MP to assess the complex construct as a central component of physical development should be implemented considering its high importance for health. A common and widely accepted MP monitoring tool would allow a pooling of data from different studies, and the lack of nationally and internationally representative samples could be addressed. This would open new insights and possibilities of comparing MP globally and over several decades (15). In addition, it is necessary to establish age and gender-adjusted reference values, and to provide normative criterion values of adequate health-influencing MP for standardized national and international monitoring (19, 25).

In Germany, the German Motor Test 6–18 (GMT 6–18) (26) and the adjusted Kinderturntest Plus 3–10 (KITT+ 3–10) (27) are widely used MP assessment tools.

In this paper, we aim to demonstrate the best practice example for a valid, objective, reliable, and economic MP tool, which targets monitoring MP levels and gives added practical value (the Fitness Barometer). The collection of data by practitioners in the direct setting of physical activity of children and adolescents establishes a collaborative relationship from which researcher, as well as the testing educators, teachers, trainers, and coaches, can extract the optimal benefits. The enormous amount of resources normally required for a long-term continuous cohort study is thus reduced, making the approach of monitoring much easier.

We show examples of which key questions can be answered within the context of the Fitness Barometer and what can be derived from these answers for practical application.

These are:

1. What is the mean value of MP in children aged 3–10 years in the German federal state of Baden-Württemberg?
2. What is the mean BMI in children aged 3–10 years in the German federal state of Baden-Württemberg?
3. Are there differences in MP in children aged 3–10 years in the German federal state of Baden-Württemberg depending on BMI?
4. Is there an influence of the COVID-19 pandemic on the MP in children aged 3–10 years in the German federal state of Baden-Württemberg?

## Materials and Methods

### The GMT 6–18 and KITT+ 3–10

The raw data of the Fitness Barometer was collected with the MP assessment tools GMT 6–18 and KITT+ 3–10. These are effective and economical MP assessment tools developed to be conducted in practical settings. The GMT 6–18 is based on the approach of Bös and Mechling (28) and KITT+ 3–10 is an adjustment for younger children. It contains eight test items representing the five main dimensions of MP endurance, strength, speed, coordination, and flexibility. Additionally, constitutional data including height, weight, and BMI were collected, and children's age and sex, as well as test date and other characteristics of data collection were recorded (28). Table 1 shows the different test items in the main dimensions.

**Table 1:** Test items of the GMT 6–18 and KITT+ 3–10.

**Dimension**	**Test item**
Endurance	6-min run
Strength	Standing long jump
	Sit-ups
	Push-ups
Speed	20-m dash
Coordination	Balancing backwards
	Jumping sideways
Flexibility	Stand and reach

### Data Collection Using GMT 6–18 and KITT+ 3–10

From 2012 to 2020, the test tools GMT 6–18 and KITT+ 3–10 were used to test MP in Germany, with a main emphasis on one federal state (Baden-Wuerttemberg). The Fitness Barometer is a project in cooperation with the Kinderturnstiftung of Baden-Württemberg and therefore drawing of the sample is limited through structural reasons on this federal state of Germany. However, Baden-Württemberg is the third largest federal state in Germany and has 704,725 children in our analyzed age-group (29). For the future an extension of the project all over Germany is planned.

The relevant target group of kindergartens, schools, and sports clubs were informed via newsletter and informational material from the ministry of education. They were invited to participate and collect data with the KITT+ 3–10 and GMT 6–18.

The implementation of the test tools is easy to conduct and can be integrated into regular physical activity sessions. Teachers, educators, trainers, and coaches were trained as multipliers and, with the help of material and additional scientific support, enabled to conduct the tests with their groups. They entered data into an evaluation software which calculates results for the subjects and group-based profiles compared with norm values (26, 27).

The raw data of the children's MP was anonymized and pooled after a quality check using the e-Research infrastructure MO|REdata (Karlsruhe Institute of Technology, Karlsruhe, Germany). To analyze the data from communities/regional councils in Baden-Wuerttemberg, only data which could be allocated to the federal state through postal code were extracted from the pooled overall data set and analyzed with IBM SPSS Statistics 27.

The first pooling in MO|REdata was conducted with the data base of raw data from 2012 until 2018, and analyzed in the first publication of “the Fitness Barometer” (30). Subsequently, this data base was extended using the actual raw data of the previous year. Therefore, this paper comprises data, collected by practitioners from 2012 until the end of 2020.

### Sample Description

The sample comprises data from children between the ages of 3 and 10. Most data derive from investigations using KITT+ 3–10 and were supplemented with age-specific data from the GMT 6–18 through data pooling. In the strict sense and with statistical objectivity the sample does not meet representativity. However, the analysis of the communities/regional councils in which the GMT 6–18 and KITT+ 3–10 were implemented shows a comprehensive distribution.

From 2012 through 2019, data from 22,930 children [MV ± SD: age: 6.68 ± 1.75; weight: 25.9 ± 7.3 kg; height: 124.7 ± 12.2 cm] from schools, kindergartens, and sports clubs were included in the analysis. Among them, 51% (*n* = 11,654) were boys and 49% (*n* = 11,276) were girls. The distribution of age groups was 28% (*n* = 6,511) 3–5 years old (kindergarten age) and 72% (*n* = 16,419) 6–10 years old (elementary school age).

During 2020, testing was difficult due to the COVID-19 pandemic conditions. Therefore, data was only collected from 934 children [MV ± SD: age: 6.75 ± 1.89; weight: 25.8 ± 7.8 kg; height: 125.2 ± 13.0 cm]. The distribution of gender was equal, and 32% (*n* = 303) were aged 3–5 years and 68% (*n* = 631) were aged 6–10 years. This latest data was pooled with the data from 2012 through 2019.

Overall, the data included in the statistical analysis of this study covers a time frame of 9 years (2012–2020), and the total number of children was 23,864 [MV ± SD: age: 6.6 ± 1.76; weight: 25.9 ± 7.3 kg; height: 124.7 ± 12.3 cm]. Among them, 51% (*n* = 12,123) were boys and 49% (*n* = 11,741) were girls. The different age groups comprised 29% ages 3–5 (*n* = 6,814) and 71% ages 6–10 (*n* = 17,050). Table 2 shows the gender-specific samples of the different investigation periods.

**Table 2:** Sample description (means ± SD).

	**2012–2019**		**2020**		**2012–2020**	
	**Male**	**Female**	**Male**	**Female**	**Male**	**Female**
*N*	11,654 (51%)	11,276 (49%)	469 (50%)	465 (50%)	12,123 (51%)	11,741 (49%)
Age	6.7 ± 1.8	6.7 ± 1.8	6.9 ± 1.9	6.6 ± 1.9	6.7 ± 1.8	6.7 ±1.8
Weight	26.2 ± 7.4 kg	25.5 ± 7.2 kg	26.4 ± 8.0 kg	25.1 ± 7.6 kg	26.3 ± 7.4 kg	25.5 ± 7.3 kg
Height	125.5 ± 12.2 cm	123.8 ± 12.2 cm	126.4 ± 13.2 cm	123.9 ± 12.8 cm	125.5 ± 12.2 cm	123.8 ± 12.3 cm

### Statistical Analysis

Statistical analyses were conducted with IBM SPSS Statistics 27. The pooled data set includes the data from the investigations conducted from 2012 through 2020. For a specific analysis of the effects and consequences of the COVID-19 pandemic, the results of the investigation period from 2012 through 2019 were compared with those from 2020.

To analyze the current levels of MP and BMI (Questions 1 and 2) compared to the nationwide reference sample, data was classified into reference percentiles, and we examine age and gender-specific relationships. A sum score describing the overall MP level was calculated from the percentile results of the test items. This sum score was only calculated if all four (age range from 3 to 5) or all eight (age range from 6 to 10) test items were completed. The representative percentile curves for Germany were used for the six test items stand-and-reach, push-up, sit-up, standing long jump, jumping sideways, and balancing backwards (31). For 20-m dash and 6-min run percentile curves were created based on the raw data of the overall data set with the data sets KITT+ 3–10 and GMT 6–18 (32). Data were differentiated by age and gender, and differences were examined with *t*- tests for independent samples and descriptive statistics after results were placed in reference percentiles. Level of significance was set at *p* < 0.05. The standardized effect size was calculated using Cohen's *d*, classifying small (0.20), medium (0.50), and large (0.80) effects (33).

BMI was calculated from individual weight and height and classified into percentiles of BMI and categorized according to the percentile groups described by Kromeyer-Hauschild (34). As we are conducting a nationwide comparison of German children, we used reference percentiles for Germany. Age and gender-specific differences were examined based on percentage frequency analysis and descriptive statistics (chi- square-test). Level of significance was set at *p* < 0.05. Cramer's *V* was used to state the power of relation and categorized into small (0.10), medium (0.30), and large (0.50) effects (33).

The relationship between fitness sum score and BMI and the short-term effects and consequences of the COVID-19 pandemic on MP (Questions 3 and 4) were examined using single analysis of variance. Effect sizes were assessed with omega square (ω^2^) due to lower bias. Limits of effect sizes were 0.01 (small effect), 0.06 (medium effect), and 0.14 (large effect) (33). In consequence of homogeneity of variance, the Hochberg correction was applied for *post-hoc* multiple comparisons. The Welch test was used when homogeneity of variance was not given and the Games- Howell correction was used as *post-hoc* test. Level of significance was set at *p* < 0.05. Confidence intervals were also stated. The additional information of effect sizes and confidence intervals of the mean values were stated in order to be able to assess the relevance of the differences more objectively. With our sample size of more than 20,000 participants, even small differences in the mean values result in significance.

## Results

### Motor Performance in Tested Children (Question 1)

Of the 23,864 children tested, *n* = 19,655 (= 82.4%) completed all test items in their age group and as a consequence was computed the sum score of MP. Overall, the children reached a mean value of 57.03 (*SD* = 18.85). The nationwide reference value is 50 (mean); this implies that MP of the sample from Baden-Wuerttemberg was 7% better compared to the national average for Germany (31).

The comparison of gender specific differences showed that boys (mean = 58.60, *SD* = 18.59) scored three percentile ranks better than girls (mean = 55.43, *SD* = 18.98), but the effect size revealed small practical relevance [*t*_(19,653)_ = 11.86, *p* < 0.001, *d* = 0.20].

The level of MP did not differ significantly between the specific age groups: kindergarten age (mean = 56.87, *SD* = 21.23) and elementary school age [mean = 57.09, *SD* = 17.85, *t*_(8,599)_ = −0.68, *p* = 0.499].

### BMI in Tested Children (Question 2)

BMI-categorization according to Kromeyer-Hauschild (34) was carried out for *n* = 23,656 (99.1%), and classified 12.7% (*n* = 2,990) of the tested children as overweight or obese. This 12.7% could be divided into 5.2% (*n* = 1,225) obese and 7.5% (*n* = 1,765) overweight children. 79.2% (*n* = 18,725) children were normal weight, and 8.2% (*n* = 1,941) were underweight.

Chi-square tests analyzing the relationship between BMI category and gender across the general sample were significant but with no relevant differences [$\chi_{\left(4\right)}^{2}$ = 19.25, *p* = 0.001, Cramer *V* = 0.03; *n* = 23,656]. However, the boys in the sample were 0.6% more overweight than the girls (boys: 12.9% *n* = 1,555; girls: 12.3% *n* = 1,435).

**Figure 1:**
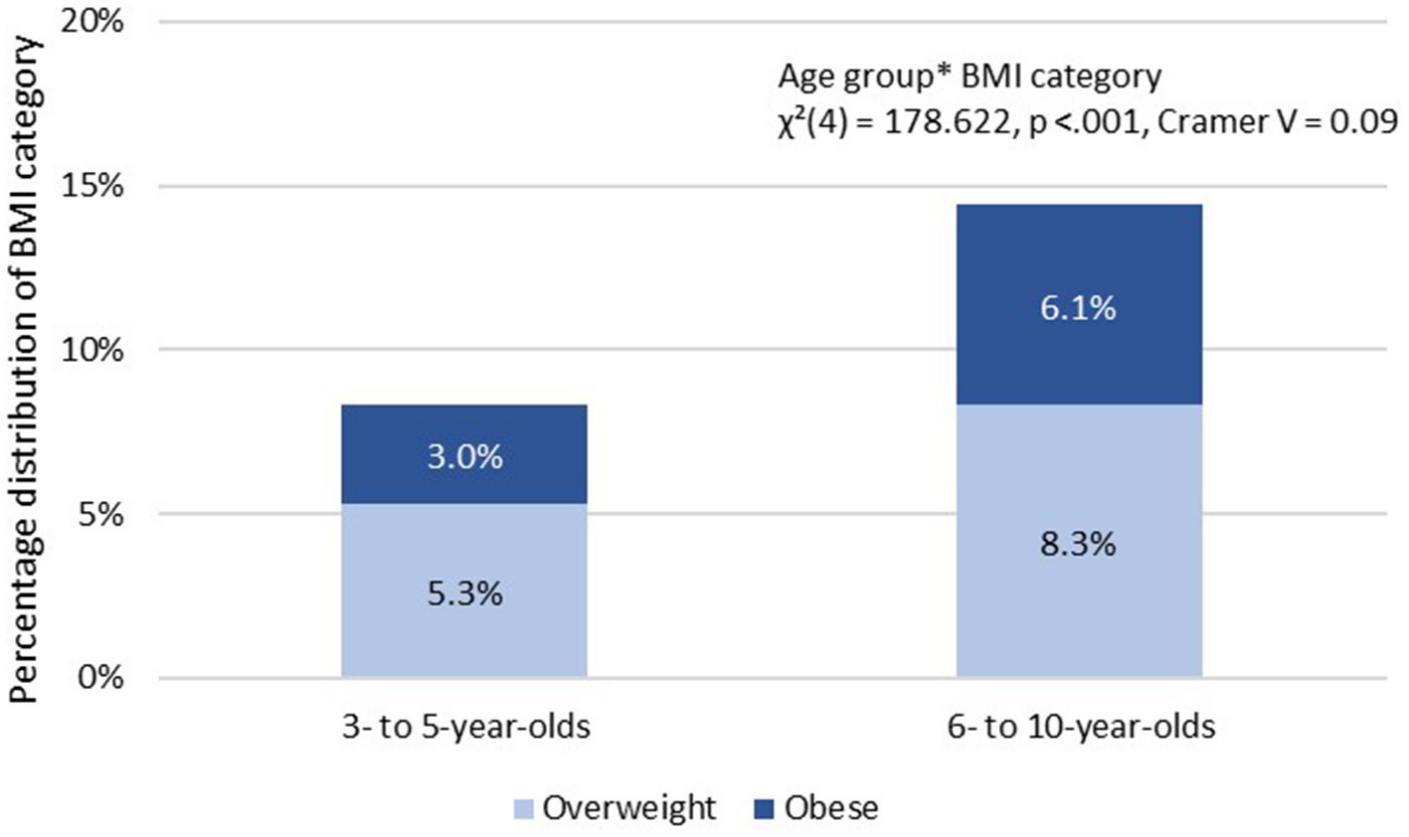
Percentage distribution of overweight and obese within BMI category overweight between age groups.

Comparing age group-specific differences, overweight increased from 8.3% at kindergarten age (3- to 5-year-olds, *n* = 557) to 14.4% at elementary school age (6- to 10-year-olds, *n* = 2,433). The percentage of obese children in the sample doubled from 3.0% (kindergarten age) to 6.1% (elementary school age). Underweight was nearly the same in both age groups, but normal weight decreased from 83.8% (3- to 5-year-olds, *n* = 5,675) to 77.3% (6- to 10-year-olds, *n* = 13,050). Chi-square-tests reveal significance between BMI category and age group with a small effect [$\chi_{\left(4\right)}^{2}$ = 178.62, *p* < 0.001, Cramer *V* = 0.09; *n* = 23.656]. Figure 1 illustrates the percentage distribution of overweight and obese within BMI category between age groups.

### Relationship Between MP and BMI in Tested Children (Question 3)

The relationship between BMI categories and mean value of MP was examined for 81.8% (*n* = 19,528) of the children tested.

There was a significant relationship between BMI category and mean value of MP [*F*_(4,19,523)_ = 224.81, *p* < 0.001] with small effect size ω^2^ = 0.04. Normal weight children achieved the highest fitness percentile values.

The *post-hoc* analysis revealed that fitness sum score of the BMI category obese was significantly different (*p* < 0.001*)* from all other categories.

Obese children scored 16 percentile ranks lower (−16.04, 95% CI [−17.74, −14.35]) than normal-weight children in the tested sample and 7.8 percentile ranks lower than overweight children (−7.80, 95% CI [−9.92, −5.67]). Anyway, overweight children had a significantly lower fitness sum score than normal weight children (−8.25, 95% CI [−9.65, −6.85]). Table 3 shows the mean values of MP in the different BMI categories.

**Table 3:** Mean value of MP in the different BMI categories.

	**BMI category**				
	**≤3**	**>3–10**	**>10–90**	**>90–97**	**>97**
*N*	494	1,087	15,542	1,448	957
Mean value of MP (95% CI)	55.44 (53.82–57.05)	57.75 (56.64–58.87)	58.48 (58.19–58.77)	50.23 (49.30–51.17)	42.44 (41.30–43.58)
			*[F_(4,19,523)_ = 224.81, p < 0.001, ω*^2^ *= 0.04]*		

### Motor Performance and the COVID-19 Pandemic (Question 4)

In 2020, *n* = 934 children were tested. Of these, 79.3% (*n* = 741) completed all test items in their age group and a sum score of MP was conducted. The children tested in the period from 2012 through 2019 reached a mean value of 56.95 (*SD* = 18.84, *n* = 18,914) and those tested in 2020 reached a mean value of 59.17 (*SD* = 19.07, *n* = 741).

Comparing the mean values of the main dimensions between the period from 2012 through 2019 and 2020, there was a significant decrease of 3.7 percentile ranks for endurance [2012–2019: mean = 49.96, *SD* = 28.13, *n* = 15.337; 2020: mean = 46.27, *SD* = 29.79, *n* = 540; Welch's *F*_(1,573)_ = 8.08, *p* = 0.005]. Similarly, for speed, the percentile rank decreased from mean of 49.79 for 2012–2019 (*SD* = 28.73, *n* = 15,526) to mean of 42.80 in 2020 (*SD* = 27.13, *n* = 564) and the difference was significant [Welch's *F*_(1,610)_ = 35.92, *p* < 0.001]. However, the effect sizes did not measure practical relevance.

The percentile rank of strength, representing the test items standing long jump, sit-ups, and push-ups, increased significantly in 2020 (mean = 61.80, *SD* = 27.52, *n* = 825) compared to the period from 2012 to 2019 [mean = 56.11, *SD* = 26.48, *n* = 20,950; ANOVA *F*_(1,21,773)_ = 36.44, *p* < 0.001]. There was also a 2.5 percentile rank increase in the flexibility percentile [2012–2019: mean = 50.60, *SD* = 31.57, *n* = 21,140; 2020: mean = 53.06, *SD* = 30.12, *n* = 884; Welch's *F*_(1,966)_ = 5.62, *p* = 0.018], but without practical relevance.

**Figure 2:**
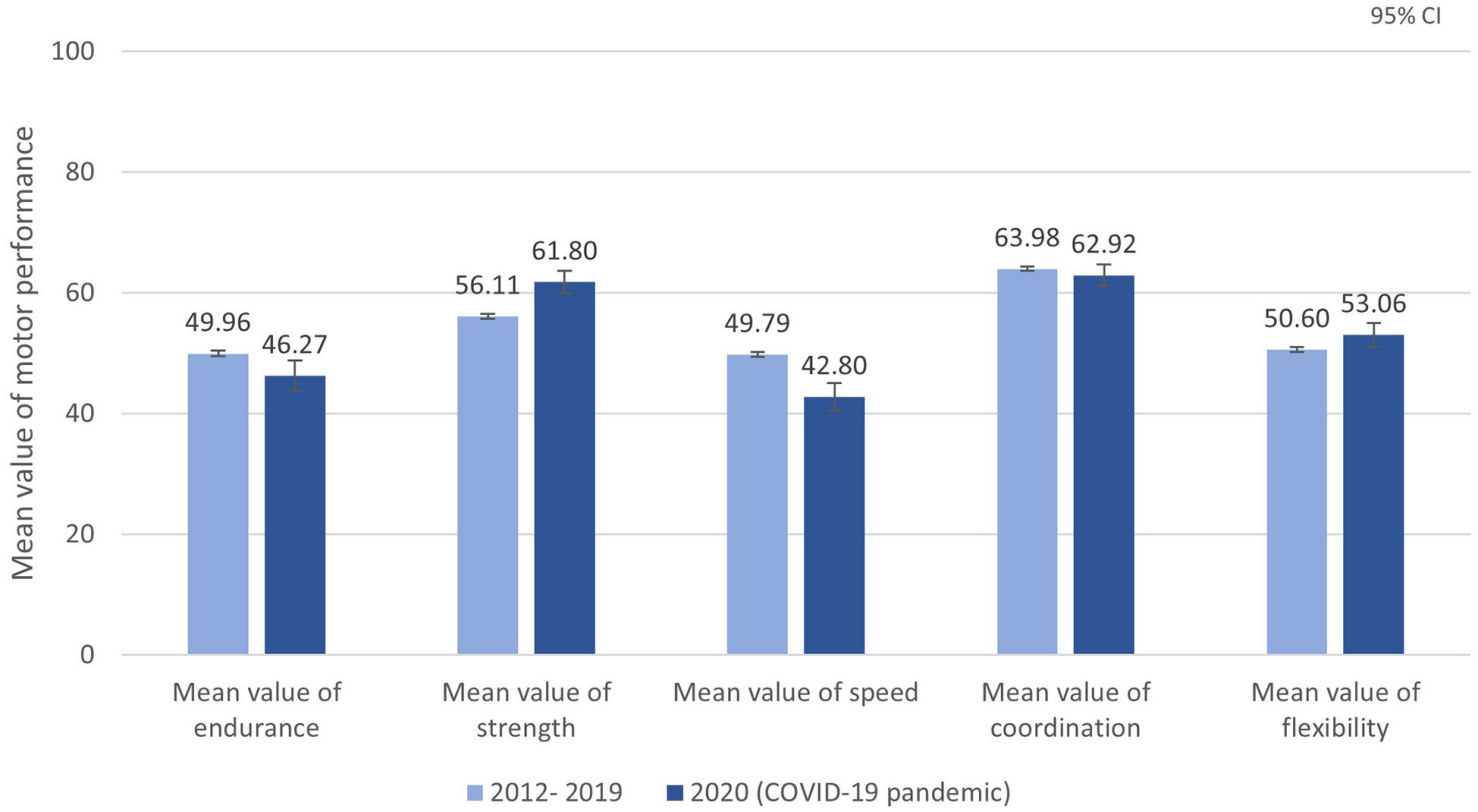
Mean values of MP for the five dimensions according to field testing period.

There was no statistically significant difference between the coordination percentile from 2012 to 2019 (mean = 63.98, *SD* = 31.57, *n* = 21,140) compared to the coordination percentile in 2020 [mean = 62.92, *SD* = 26.00, *n* = 856; ANOVA *F*_(1,22,014)_ = 1.461, *p* = 0.227]. Figure 2 shows the mean values of MP for the five dimensions according to field testing period.

## Discussion

The aim of this paper was to demonstrate a concrete best practice example of a valid, objective, reliable, and economical MP tool (the Fitness Barometer) that is implemented in practice and provides benefits to practitioners and researchers. Furthermore, we show which relevant key questions can be answered in the context of the Fitness Barometer and which consequences can be derived from this for practical implementation.

The analysis of MP in children tested in one federal state of Germany (Question 1) revealed that they were fitter compared to the national reference group and that boys scored at a higher percentile than girls. With a mean value of 57.03, the MP is average and matches results of the MoMo study, which is representative for Germany (31).

In our sample, one-eighth of the children tested were overweight or obese (Question 2). Cross-sectional results from the KIGGS-study for the period 2014–2017 show a prevalence of overweight and obesity of 15.4% for ages 3–17 years (35). They also found evidence for an increase in both overweight and obesity with increasing age, confirming our findings that the proportion of obesity doubles between kindergarten and elementary school. This significant prevalence is even more dramatic when one recognizes that this period is a fundamental and predeterminate for the future development of MP (3, 10, 36, 37). Therefore, interventions and programs should target this transition and the specific changes that occur in the daily routines of children's lives. For example, schools and institutions should establish movement breaks in the classroom and create an environment and infrastructure which invites to move and to be physical active.

The detected relationship between MP and BMI category (Question 3) shows the significance of specific support for overweight children. With the present finding and several evidence that they have poorer MP compared to normal weight children, it is crucial to break the cycle. There are subsequently more consequences and causalities of overweight and obesity leading to an inactive lifestyle (13, 14, 37). The immediate practical suggestion is to create situations giving overweight children the possibility of using their body mass positively and experience motivation and enhancement.

The main findings of the analyses of the impact of the COVID-19 pandemic on MP (Question 4) pointed out to the relevant role of physical activity with peers and within an institution (38). While the percentiles in the dimensions strength and coordination increased and flexibility remained stable, endurance, and speed percentiles decreased significantly. Even though these are only short-term tendencies and long-term effects must be examined in further analyses, it seems that some alternative options like online and indoor workouts for children may mitigate some dimensions during the COVID-19 pandemic, but not when running and sprinting are essential. The results of an investigation within the MoMo study showed that daily activity increased during the lockdown, but physical activity still decreased (38). It strengthens the role of physical activity in an organized form and setting like sports clubs, schools, or in kindergartens. Intensity is higher and the quality of movement is better through trained and qualified coaches. For future comparable situations of massive restrictions, opportunities and concepts of possible adequate activity and MP should be developed to mitigate the effects of missing organized sports and physical activity for everyone and to build awareness of the consequences of insufficient physical activity (39). It will be interesting to investigate the long-term effects and influence of the COVID-19 pandemic in so-called “Corona- age groups” and makes the monitoring of MP even more necessary. With the investigation period of the Fitness Barometer since 2012 we have cohort data before and after the COVID-19 pandemic and therefore an innovative and rare possibility of comparison.

The constituted results of the statistical analysis give an overview of the aspects of monitoring MP within the Fitness Barometer. First, it points out the current and latest state of MP in children and adolescents continuously. Second, changes and trends were identified, and finally with regard to the large sample displaying the relevant age-group, policy, and decision-maker are influenced and suggested to initiate specific and differentiated concepts of promotion of MP in children and adolescents.

As requested by Lopes et al. (19), the Fitness Barometer fulfills the requirement of a multidisciplinary approach in many ways. The special aspect of data collection with the GMT 6–18 and KITT+ 3–10 through practitioners guarantees a field-based assessment in various settings. That is why, large sample sizes within different age groups and cultural contexts are possible. Test items are fundamental tasks which make the latent construct of MP measurable. Standardization is necessary for national and international comparability. To ensure standardization, there is a detailed manual with precise descriptions and additional information on the homepage (www.turnbeutelbande.de). In addition, multipliers were trained by experts and a service hotline was set up. An English version of the GMT 6–18 manual is in progress and will be published soon under the name IPPTP-R (40).

The research confirms objectivity and standardization of the test items with no problems regarding test implementation. Test-retest reliability is satisfactory but reveals the difficulties in measuring coordinative abilities. Precise instructions and explanations are essential, and instructions for administering the test should be adhered strictly to ensure high reliability (26). Overall, the analysis of construct validity confirms the quality of the theoretical framework based on the GMT 6–18 and the assumption of dimensionality of motor performance (41). In particular, with an investigation of expert ratings and their assessment of good practicability of the GMT 6–18, the challenge of serving research and practice mentioned by Lopes et al. (19) is overcome. The test tool meets the research criteria and yet is economical and efficient in its implementation.

With many small samples from a large number of testing events in kindergartens, schools, and sports clubs, pooling data allows MP development to be monitored based on a large data base. For the Fitness Barometer, we use the MO|REdata e-research infrastructure and ensure the inclusion of all available data (www.motor-research-data.org). The collaborative repository for MP data was developed to store, combine, and evaluate data. It provides a global and robust overview of specific test items to state a comprehensive prevalence and monitoring of trends over time (42).

The Fitness Barometer with its base of pooled data gives an example of a feasible monitoring system. The widespread test tools (GMT 6–18 and KITT+ 3–10) guarantee a consistent and standardized investigation and collection of data. Regular and consistent monitoring with standardized methods should be implemented into the life of educational institutions at the national level by decision-makers and in policy (19).

This integration of relevant stakeholders provides awareness and acceptance by influencing networks and is essential for evidence that leads to practical implementation and programs targeting children's MP (19). The Fitness Barometer is a cooperation between many partners within their specific expertise and perspectives, e.g., sports clubs, sports associations, health promotion foundations, health insurance companies. The common aim is to increase the awareness about the importance of health-related physical activity in childhood. With the Fitness Barometer, practical recommendations, and guidelines based on evidenced monitoring data of MP were published annually to effectively promote physical activity. This fulfills the need to translate research into practice. For example, the data and findings of the Fitness Barometer collection could be reused for political initiatives and reports for the general public like the global alliance of “active healthy kids,” an initiative which develops report cards on physical activity of youth in different countries (43).

## Conclusion

Given the known benefits of high MP levels for active lifestyles and health, the importance of promoting physical activity in childhood and adolescence becomes clear. To adjust and assess programs, interventions, and initiatives, consistent scientific monitoring with findings from standardized assessment tools is the basis on which adequate measures should be built. Public awareness and a common effort to promote the need for physical activity, especially in this stage of life, is the main factor for effective implementation. The Fitness Barometer is a best practice example for monitoring MP with pooled data collected from practitioners.

## Data Availability Statement

The datasets presented in this study can be found in online repositories. The names of the repository/repositories and accession number(s) can be found at: http://motor-research-data.org/.

## Ethics Statement

The studies involving human participants were reviewed and approved by Karlsruhe Institute of Technology. Written informed consent to participate in this study was provided by the participants' legal guardian/next of kin.

## Author Contributions

TE drafted the initial manuscript, did the statistical analysis, and revised the manuscript. CN contributed particularly to the Introduction and Discussion sections. CN and KB reviewed the manuscript and lead the project. All authors have read and agreed to the published version of the manuscript.

## Conflict of Interest

The authors declare that the research was conducted in the absence of any commercial or financial relationships that could be construed as a potential conflict of interest.

## Publisher's Note

All claims expressed in this article are solely those of the authors and do not necessarily represent those of their affiliated organizations, or those of the publisher, the editors and the reviewers. Any product that may be evaluated in this article, or claim that may be made by its manufacturer, is not guaranteed or endorsed by the publisher.

## Footnotes

^1^There are several different terms, definitions, and notions of the complex and multi-dimensional construct of children's motor performance (MP). In this paper, we use MP as the general level of someone's ability to utilize motor skills, fundamental movement skills, and motor competence. With an increasing level of MP, the essential fundamental competence and knowledge to be physically active is given (2–4).
